# Design and Implementation of a RF Powering Circuit for RFID Tags or Other Batteryless Embedded Devices

**DOI:** 10.3390/s140814839

**Published:** 2014-08-13

**Authors:** Dongsheng Liu, Rencai Wang, Ke Yao, Xuecheng Zou, Liang Guo

**Affiliations:** School of Optical and Electronic Information, Huazhong University of Science & Technology, Wuhan 430074, China; E-Mails: dsliu@mail.hust.edu.cn (D.L.); keyao@hust.edu.cn (K.Y.); estxczou@gmail.com (X.Z.); guolianger@gmail.com (L.G.)

**Keywords:** batteryless embedded devices, wireless sensor network, RFID tag chip, rectifier, regulator

## Abstract

A RF powering circuit used in radio-frequency identification (RFID) tags and other batteryless embedded devices is presented in this paper. The RF powering circuit harvests energy from electromagnetic waves and converts the RF energy to a stable voltage source. Analysis of a NMOS gate-cross connected bridge rectifier is conducted to demonstrate relationship between device sizes and power conversion efficiency (PCE) of the rectifier. A rectifier with 38.54% PCE under normal working conditions is designed. Moreover, a stable voltage regulator with a temperature and voltage optimizing strategy including adoption of a combination resistor is developed, which is able to accommodate a large input range of 4 V to 12 V and be immune to temperature variations. Latch-up prevention and noise isolation methods in layout design are also presented. Designed with the HJTC 0.25 μm process, this regulator achieves 0.04 mV/°C temperature rejection ratio (TRR) and 2.5 mV/V voltage rejection ratio (VRR). The RF powering circuit is also fabricated in the HJTC 0.25 μm process. The area of the RF powering circuit is 0.23 × 0.24 mm^2^. The RF powering circuit is successfully integrated with ISO/IEC 15693-compatible and ISO/IEC 14443-compatible RFID tag chips.

## 1. Introduction

The applications of RFID technologies, including supply chain management, access control to buildings, public transportation, airport baggage handling, express parcel delivery and logistics, have increased rapidly in recent years [1–3]. Owing to its wireless and far field measurement capabilities, the combination of RFID technology and wireless sensing networks has also found a large number of applications such as environmental monitoring and healthcare devices [4–6]. RFID tags are often classified as passive or active. The passive tag is powered by an electromagnetic RF (radio frequency) wave transmitted by a reader, while the active tag is powered by a battery. The passive tag has advantages of low cost and long life, thus enable sensing nodes in a sensing system to be placed in positions that are not easily accessible [7,8].

However, passive RFID tags, and other batteryless embedded devices such as contactless smart cards, wireless sensor nodes and implantable biomedical devices, have to work at a considerably long distance from the transmitter [9,10]. Moreover, power converted from the electromagnetic RF wave to the tag should be sufficient to supply device in its operating distance, especially when a power hungry sensor is integrated. As the RF energy received by the tag (transponder) or other batteryless embedded devices decreases rapidly with distance, the induced voltage across the tag antenna is often variable in big interval and sensitive to temperature and process variation [11,12].

Thus, key challenges to implement a passive tag are harvesting energy with high efficiency and generating a stable voltage source for a digital baseband controller, the memory of the tag and its functional parts. This voltage source must be immune to process, input voltage and environmental temperature changes. Meanwhile, in order to meet the requirements for a more complicated RFID tag such as integrating sensors and CPU in a tag chips, the RF power circuit should be capable of handling heavy current loads [13,14].

To solve those problems, many RF power circuit design techniques for passive RFID tags have been reported to improve the power conversion efficiency and stability of the regulator power supply [14–17].

A low power RF harvester for a smart passive sensor tag used in structural health monitoring applications was proposed in [16]. The RF harvester harvests energy from the RF field and provides a stable and controllable power supply. Measurements of this design indicate full operation of the sensor tag for time intervals of 20 ms for up to 4 m distances with a tag reader power of 200 mW. However, this RF harvester is a chipless design while tags that utilize an application-specific chip are an undeniable trend for passive tag applications.

For specific tag chip designs, a low power CMOS voltage regulator for a wireless blood pressure biosensor is presented in [8]. A low voltage regulator, as part of the associated electronics of a blood pressure bio-medical implanted device, provides the power supply for the system. The line regulation of this regulator is 39 mV/V and its total power consumption is 1.2 mW. Since the body temperature is kept stable at approximately 37 °C, the regulator of this design is not optimized for temperature changes.

Moreover, to improve the stability of the power supply, a RF powering circuit which is immune to variations of temperature and voltage is proposed in [14]. A ripple stabilizer and a temperature stabilizer have been integrated to maintain a stable reference voltage that is insensitive to temperature variation and voltage ripples. Its line regulation is 12 mV/V and temperature rejection ratio is 0.2 mV/°C. However, the two huge resistors used for meeting the requirement of the low static current occupy a large area.

As for achieving high PCE, a RF to DC CMOS rectifier with high efficiency over a wide input power range for RFID applications is implemented in [17]. The rectifier is based on a novel active load circuit which adjusts the output current as a function of the incoming RF power. This design allows maximizing both the efficiency and sensitivity of the circuit and achieves a highest PCE of 45%.

In this paper, a RF powering circuit which is immune to process, voltage and temperature changes with high PCE is designed for a HF passive RFID tag chip. Compared with previous works, the input voltage of the regulator ranges from 4 V to 12 V which is much larger than that reported in the previous works, while a stable output voltage is also maintained against temperature and voltage variations. Furthermore, a rather high PCE is achieved in this work, which means this RF powering circuit can provide more power to the tag and hence enable further identification distance and it can be applied in more complicated passive tags.

The paper is organized as follows: Sections 2 and 3 describe the design and implementation of the RF powering circuit. The simulation and measurement results are presented in Section 4 and followed by conclusions in Section 5.

## 2. Architecture of the RF Powering Circuit

A passive RFID tag generally consists of an analog front end (AFE), a digital baseband and an EEPROM, where the EEPROM can be divided into a logic control part and a memory part. If the tag is integrated with other specific functions such as sensing or monitoring, a functional block can be added to the tag system. In this design, the digital baseband and the logic control part of EEPROM are realized with standard cells, while the analog design is responsible for the AFE and the memory part of EEPROM.

**Figure 1.**
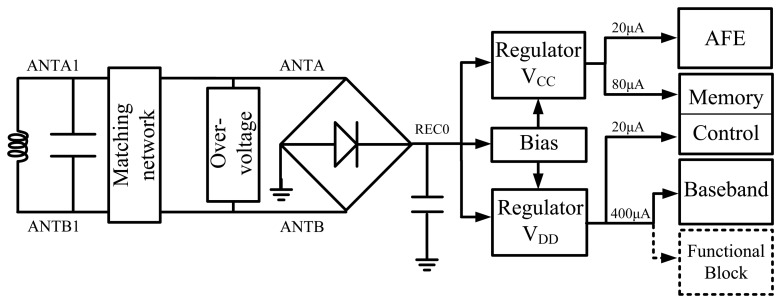
Structure of the RF powering circuit.

As shown in Figure 1, the RF powering circuit comprises four parts. First, the energy harvesting circuit, consisting of an inductor and a resonance capacitor, harvests energy from an electromagnetic field through inductive coupling and resonance. A magnetic coil is used as antenna in this design. Then, the rectifier circuit, which consists of a bridge rectifier and a capacitor, converts the input RF signal from electromagnetic field into a DC signal with ripples. Consisting of a bias circuit and regulator V_CC_/V_DD_,the regulator is designed to convert the DC signal with ripples into a stable power supply. The output voltage of the regulator has much smaller ripples and can drive several loads. Finally, an over-voltage protection circuit is presented to limit voltage of antennas by bleeding spare energy in antennas into the ground and this ensures that the whole chip has a stable and reliable operation.

The regulator V_CC_ drives the AFE and the memory part of the EEPROM. The average load current of the regulator V_CC_ is 100 μA, in which 80 μA is for the memory array and 20 μA is for the AFE. The regulator V_DD_ drives the digital baseband controller and the logic control of the EEPROM. The load current of the regulator V_DD_ is 420 μA, where 20 μA is for the EEPROM logic control, and 400 μA is for baseband controller and other functional blocks.

In our design, the digital and analog circuits are driven by V_DD_ and V_CC_ respectively, which makes it flexible to design the driving capability for different circuits, therefore meeting the requirements of high power consumption chips such as digital baseband controllers based on FSM, security chips, CPUs and chips integrated with sensors. Meanwhile, the huge noise of the digital circuit can be isolated by driving the digital and analog part separately. Thus the cost of noise restriction in the AFE can be minimized in this way.

## 3. Design and Implementation of RF Powering Circuit

### 3.1. Rectifier Circuit

**Figure 2.**
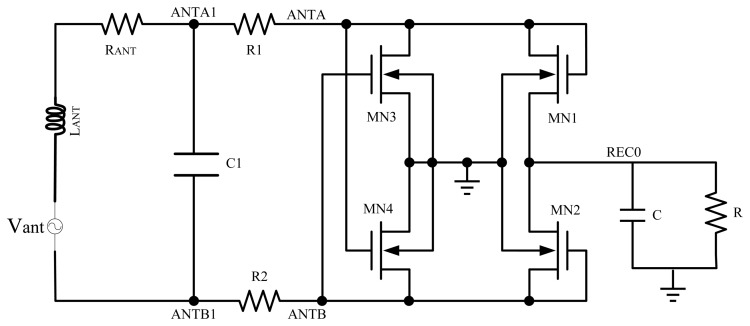
NMOS gate cross-connected bridge rectifier.

As shown in Figure 2, a NMOS gate cross-connected bridge rectifier structure, which is usually used in RF transponders for its high PCE, is adopted as the rectifier circuit.

In Figure 2, the gates of NMOS transistors MN3, MN4 are cross connected to the antennas and MN1, MN2 are connected as diodes. The antenna can be equivalent to an AC voltage source *V_ant_*, an inductor *L_ANT_* and the parasitic resistor *R_ANT_*. Resistors R1, R2 form the matching circuit, which matches the impedances of the antenna and the rectifier, thus *L_ANT_* and C1 resonate and the maximum voltage values of ANTA and ANTB will be obtained. The impedance of R1 and R2 is obtained according to the input impedance of the cascading circuit to the resistors R1 and R2.

**Figure 3.**
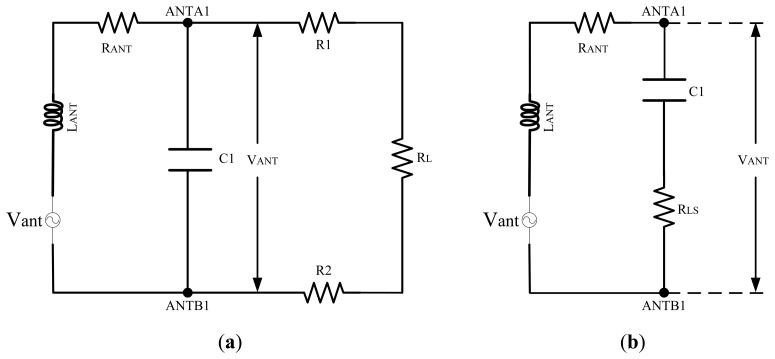
(**a**) Equivalent circuit of the NMOS gate cross-connected bridge rectifier; (**b**) Equivalent circuit of (a) after parallel to serial impedance conversion.

The equivalent circuit of the NMOS gate cross-connected bridge rectifier is shown in Figure 3a. Resistors R1, R2 form the matching circuit, and *R_L_* is the equivalent input impedance of the cascading circuit to the resistors R1 and R2, *V_ANT_* is the input voltage of the rectifier. Resistors R1, R2, and *R_L_* are connected to C1 in parallel. They can be equivalently transformed to be connected in series as shown in Figure 3b. In Figure 3b, *R**_LS_* is obtained after the parallel to serial impedance conversion of resistors R1, R2, and *R_L_*. An approximative value of *R_LS_* is calculated by the equation below:

(1) $$R_{\text{LS}}=\frac{1}{\left(R_{L}+R_{1}+R_{2}\right)\cdot {\left(\omega C1\right)}^{2}}$$

According to the series resonance theory, the quality factor *Q* of the circuit in Figure 3b can be obtained by:

(2) Q=1ωC11(R1+R2+RL)·(ωC1)2+RANT

When the value of *Q* is greater, the input voltage *V_ANT_* will be higher. According to Equation (2), to make *Q* bigger, the values of R1 and R2 should be big as well. As the value of *R_ANT_* is 20 Ω, the value of C1 is 16.5 pF, so the values of resistors R1 and R2 are both designed to be 15.7 kΩ to make the previous factor of the denominator of Equation (2) infinitesimally smaller than the following one.

When ANTA is high and ANTB is low, MN1, MN4 are on and MN2, MN3 are off. The capacitor is charged through path ANTA-MN1-C-GND-MN4-ANTB. When ANTB is high and ANTA is low, the capacitor will be charged through path ANTB-MN2-C-GND-MN3-ANTA.

In this circuit, the threshold voltage loss is as low as a *V_TH_*, which is the threshold voltage of NMOS. As MN3 and MN4 do not produce any *V_TH_* loss, the threshold voltage loss is produced by MN1 and MN2. It can be calculated that the minimum operation input level is slightly higher than *V_TH_* because of the substrate effect. Thus, the minimum operation input level is efficiently reduced in this design. According to the preceding analysis, the antenna voltage is similar to a sinusoidal signal, and the output voltage level is [18]:

(3) $$V_{\text{REC}0}=V_{\text{ANT}}/\sqrt{2}-V_{\text{TH}}$$

where *V_REC0_* is the output voltage of the rectifier, *V_ANT_* is the peak amplitude of the antennas voltage, *V_TH_* is the threshold voltage of NMOS. As *V_SB_* = *V_REC0_*, *V_SB_* is the source-bulk voltage of MN1 and MN2, according to the bulk effect model of NMOS, there is:

(4) $$V_{\text{TH}}=V_{\text{TH}0}+\gamma (\sqrt{\left|2\phi_{F}+V_{\text{REC}0}\right|}-\sqrt{2\phi_{F}})$$

where V_TH0_ is the intrinsic threshold voltage, γ is the body effect coefficient, ϕ_F_ is the Fermi potential. According to Equations (3) and (4), *V_REC0_* increases with respect to *V_ANT_*. As the electromagnetic field strength changes from 150 mA/m to 5 A/m, which is defined in ISO/IEC 15693 standard and related ISO/IEC 10373-7 test standard, the output voltage of the rectifier varies a lot from 4 V to 12 V according to the simulation. As a rectifier converts a RF signal of antenna to a DC voltage, higher PCE of the rectifier means more energy is converted to the tag. PCE is calculated by the equation below:

(5) $$\text{PCE}=\frac{P_{\text{out}}}{P_{\text{in}}}$$

PCE can be also calculated by *PCE* = *P_out_*/(*P_out_* + *P_mos_*), where *P_mos_* is the power consumption of the transistors of the rectifier. *P_mos_* can be calculated by, *P_mos_* = *P_dynamic_* + *P_static_*, where *P_dynamic_* is the power consumption of the transistors while the transistors are on, and *P_static_* is the power consumption of the transistors while they are off. $P\text{dynamic}=\bar{V\text{on}\cdot I\text{on}}$, *V_on_* is the forward voltage drop of the transistors, and *I_on_* is the forward current of the transistors. $P_{\text{static}}=\bar{V_{\text{reverse}}\cdot I_{\text{reverse}}}$, *V_reverse_* is the reverse voltage of the transistors, and *I_reverse_* is the reverse leakage current of the transistors. The turn-on voltage of the rectifier determines the minimum input-voltage of the rectifier, higher turn-on voltage means higher Von, and greater reverse leakage current means greater *P_static_*, so while the turn-on voltage is higher, and the reverse leakage current is greater, then the input power will be greater.

According to the aforementioned analysis of resistors R1 and R2, the resistors make PCE higher. As proper R1 and R2 bring a biggest *Q* of the circuit, so the value of *V_ANT_* will be maximum when *L_ANT_* and C1 resonate. Bigger *V_ANT_* means greater *P_out_*, then the value of PCE will be higher.

As shown in Table 1, PCE is greatly influenced by the size of the MOS device. According to the ISO/IEC 15693 standard and the related ISO/IEC 10373-7 test standard, the input voltage of the rectifier is always higher than 4 V, so the *V_ANT_* is set to be 6 V as a normal value in this simulation. The load current of this RF powering circuit is less than 600 μA, and the output power of the antenna is normally less than 5 mW, so the *R_load_* is set to be 6 kΩ. In this table, MOS devices have the same width/length (represented as W/L afterwards) ratio and M is the multiplier of a MOSFET. Greater *M* value means bigger size of a MOSFET. Bigger size of a rectifier MOSFET brings smaller channel conduction resistor, thus the output voltage of the rectifier is increased and higher PCE is achieved. However, bigger size of a MOSFET increases Ids of the MOS device, hence it consumes more power. As a result, when *M* > 20, the increase of PCE becomes slower than that when *M* is smaller. Tradeoffs between PCE and power consumption should be considered when designing the rectifier.

### 3.2. Linear Regulator

**Figure 4.**
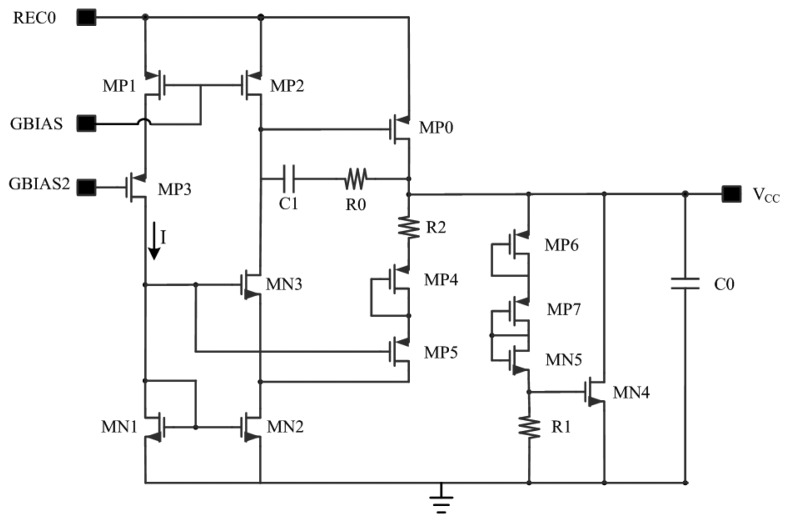
Circuit of V_CC_ regulator.

The V_CC_ voltage regulator is shown in Figure 4. By using a combination resistor and a calculation strategy for the device parameters, an optimization for regulator performance related to voltage and temperature is achieved in this work. As can be seen in Figure 4, NMOS transistors MN1 and MN2 form a current mirror. The size of MP1 is two times that of MP2. The gates of MP5 and MN3 are connected. Capacitance C1 and resistor R0 are frequency compensation devices. MP0 is a large pass transistor which enables heavier load capacity. MP4, MP5 and R2 stabilize the *V_CC_* voltage level while C0 is used for filtering and storing energy. The voltage limiter of this circuit is composed of MP6, MP7, MN5, R1 and MN4.

If calculating the output voltage through the way R2-MP4-MP5-MN1, the output voltage of the regulator in Figure 4 is given by [13]:

(6) $$V_{\text{CC}}=V_{\text{SG},\text{MP}4}+V_{\text{SG},\text{MP}5}+I_{D,\text{MP}5}R_{2}+V_{\text{SG},\text{MN}1}$$

where MP4, MP5 and MN1 are in saturation region with the same *W*/*L* ratio, according to the V-I characteristic of MOSFET in saturation region, the following equation can be given as:

(7) $$V_{\text{SG},\text{MP}5}=V_{\text{SG},\text{MP}4}=\left|V_{\text{THP}}\right|+\sqrt{\frac{2I_{D,\text{MP}5}}{\mu_{P}C_{\text{ox}}{\left(W/L\right)}_{\text{MN}1}}}$$

(8) $$V_{\text{SG},\text{MN}1}=V_{\text{THN}}+\sqrt{\frac{2I_{D,\text{MN}1}}{\mu_{N}C_{\text{ox}}{\left(W/L\right)}_{\text{MN}1}}}$$

*V_THN_* and *V_THP_* are the threshold voltages of NMOS and PMOS respectively.

As the size of MP1 is two times that of MP2, MN1 and MN2 are mirror transistors, and the current can be represented as:

(9) $$2I_{D,\text{MP}5}=2I_{D,\text{MP}2}=I_{D,\text{MP}1}=I_{D,\text{MN}1}$$

Equation (6) can be rewritten as:

(10) $$V_{\text{CC}}=\left|V_{\text{THN}}\right|+2\left|V_{\text{THP}}\right|+2\sqrt{\frac{2I_{D,\text{MP}1}}{\mu_{P}C_{\text{ox}}{\left(W/L\right)}_{\text{MN}1}}}+\sqrt{\frac{2I_{D,\text{MP}1}}{\mu_{N}C_{\text{ox}}{\left(W/L\right)}_{\text{MN}1}}}+\frac{1}{2}I_{D,\text{MP}1}R_{2}$$

In Equation (8) *I_D,MP1_* is determined by the bias circuit with the value of 2 μA. When ignoring the changes of temperature, the first and second term at the right side of Equation (10) are constant, whereas the last three terms are the only part that can be changed in this equation. Suppose that the last three terms of Equation (10) are equal. That is:

(11) $$2\sqrt{\frac{2I_{D,\text{MP}1}}{\mu_{P}C_{\text{ox}}{\left(W/L\right)}_{\text{MN}1}}}=\sqrt{\frac{2I_{D,\text{MP}1}}{\mu_{N}C_{\text{ox}}{\left(W/L\right)}_{\text{MN}1}}}=\frac{1}{2}I_{D,\text{MP}1}R_{2}=\frac{1}{3}(V_{\text{CC}}-\left|V_{\text{THN}}\right|-2\left|V_{\text{THP}}\right|)$$

According to Equation (11), the value of *(W*/*L)_MP1_* and *R_2_* can be calculated under typical process conditions. The output voltage of the regulator changes along with the environment temperature and the rectified voltage of REC0. When the voltage of REC0 increases, *V_SG,MP1_* and *V_SG,MP2_* are clamped by the bias circuit. Since MP3 is a common-gate connection PMOS, the voltage change of REC0 mostly turns out to be the drain-source voltage of MP3, so the variation of drain voltage of MP1 and the current mirror mismatch of MP1 and MP2 caused by channel modulation effect are reduced. Similarly, the matching of MN1 and MN2 is improved by MN3. Thus, the immunity of the regulator output to voltage changes of REC0 are improved, and according to the analysis of the bias circuit mentioned later in Section 3.3, the current I of this regulator is not easily affected by the supply voltage of REC0, so fluctuations of *V_CC_* caused by the supply voltage can be suppressed by a well-designed bias circuit.

Temperature Coefficient (TC) is an index which describes how temperature variations influence a specific value. It is mostly used to describe changes of resistance values. TC of this design is defined as Equation (12) *V_1_* and *V_2_* are voltages before and after the temperature change:

(12) $$\text{TC}=\frac{V_{2}-V_{1}}{V_{1}\cdot \Delta T}$$

By observing the right side of Equation (10)*V_THN_*, V_THP_, μ_P_C_ox_ and μ_N_C_ox_ are process parameters and their contributions to *V_CC_* are fixed when the *W*/*L* ratio is determined. TC of the fifth item 1/2I_D,MP1_R_2_ can be modulated by selecting the proper type of resistor. TC of the front four items of Equation (10) around room temperature can be obtained by simulation. According to the obtained TC, a proper type of resistor for R2 can be selected. In this way, TC of *V_CC_* is minimized around room temperature.

While types of resistors are limited in each process, the TC of resistors which can be chosen is also limited. To solve this problem, a concept of combination resistor is adopted in our design [19]. According to this concept, two resistors of different TC are chosen to compose a combination resistor. TC of *V_CC_* can be minimized by choosing a proper TC of this combination resistor. As the TC of the combination resistor is between that of the chosen resistors, the combination resistor has many more TC options than a single resistor. In general, by setting a proper voltage proportion of the first three items in Equation (8) and selecting a combination resistor appropriately, a desirable *V_CC_* voltage regulator circuit immune to temperature variations is designed in this work. Moreover, the circuit of the *V_DD_* regulator has the same structure with that of *V_CC_*, while loads of the output voltage are the main differences between the two, one can carry on the design and optimization of *V_DD_* regulator with the same methods as *V_CC_* mentioned above.

### 3.3. Bias Circuit

Shown in Figure 4, a bias circuit is designed to provide a stable current for the regulator and other circuits. Start-up part of this bias circuit is composed of PMOS transistors MP1 and MP2. In the process of starting up, if there are no transistors MP1 and MP2, the bias circuit will enter a condition that the voltage of GBIAS1 is high and the gate voltage of MN1 and MN2 is low when REC0 goes up. In this condition, there is no current in each branch of the bias circuit and the bias circuit isn't able to start to work. When starting up, the rectified voltage REC0 goes up and the voltage of GBIAS1 rises. At first, transistors MP1 and MP2 are both off and capacitor C1 is uncharged. Then MP2 turns on while REC0 goes high, thus making the voltage of GBIAS2 increase. Then transistors MN1 and MN2 turn on, reducing the drain voltage of MN2, thus making transistors MP5 and MP1 on. Capacitor C1 begins to charge through MP1. Hereafter, the bias circuit enters its normal working conditions when the upper plate of C1 is charged to REC0. Then MP2 turns off and the start-up circuit is out of operation afterwards.

**Figure 5.**
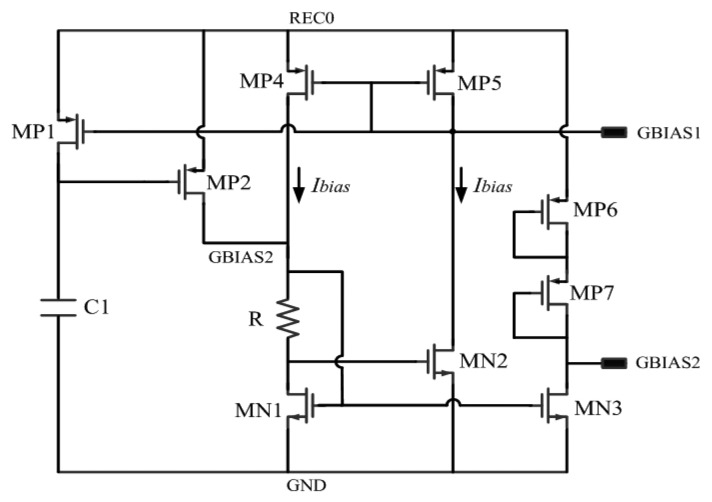
Bias circuit of RF powering circuit.

As for the bias circuit itself, the current mirror in Figure 5 is composed of PMOS transistors MP4 and MP5. The *W/L* ratio of NMOS transistor MN2 is k times of MN1. MOS devices in Figure 5 are all in saturation region. As MP4 and MP5 are mirror transistors of each other, the current of each branch (represented as *I_bias_*) is kept equal. There has the following equation [20]:

(13) $$V_{\text{GS},\text{MN}2}+I\cdot R=V_{\text{SG},\text{MP}1}$$

Since it is known that MN3 and MN4 are in saturation region, according to the V-I characteristic of MOSFET in saturation region, I_bias_ can be calculated from Equation (13):

(14) $$I_{\text{bias}}=\frac{2}{\mu_{N}C_{\text{ox}}{\left(W/L\right)}_{\text{MN}1}\cdot {R_{1}}^{2}}{\left(1-\frac{1}{\sqrt{k}}\right)}^{2}$$

Shown in Equation (14), the advantage of this bias circuit is that it can eliminate the substrate bias effect. The *W*/*L* ratio of MN1, MN2 and the resistance value of *R* can be calculated according to the requirements of power dissipation, as well as the proper selection of over-drive voltage of MN1 and MN2.

As long channel devices are used in the bias circuit, the channel length modulation effect produced by the increasing of V_REC0_ can be well suppressed. Moreover, considering the temperature response of this circuit, Equation (15) can be obtained when taking the derivative of temperature in Equation (14):

(15) $$\frac{\partial I}{\partial T}=A(\frac{1}{\mu_{N}}\cdot \frac{\partial \mu_{N}}{\partial T}+\frac{2}{R}\cdot \frac{\partial R}{\partial T})$$

While **∂μ*_N_***/**∂*T*** is a fixed process coefficient, *A* is a fixed coefficient, and *R* represents the impedance of the resistor in Figure 5. By choosing proper value of ***∂R***/***∂T***, the TC of bias current, expressed in Equation (15), can be minimized around room temperature. The combination resistor mentioned above is also adopted here.

### 3.4. Layout Optimization Methods

In this work, the RF powering circuit is designed for application in a passive RFID tag. As the tag is integrated with digital and analog parts in the same chip, the noise interference between the digital and analog parts becomes a serious problem as it operates under high frequency. As the RFID tag can work in intense electromagnetic field according to the related ISO/IEC 15693 and ISO/IEC 14443 standards, large spare energy in antennas must be bled by an over-voltage protection circuit, thus a large current is injected into the ground. At the same time, the high frequency of transient currents is conducted in the rectifier circuit.

These problems listed above can be latch-up triggers in both the analog and digital part, therefore leading to a latch-up in the chip which is a dominant failure in CMOS chip projects. Latch-up may cause a loss of data logic states or destructive failure of the whole system [21,22]. As a result, layout optimizations to isolate the noise between the analog and digital part and to reduce the possibility of failures caused by latch-up is quite essential in the layout design of this chip.

**Figure 6.**
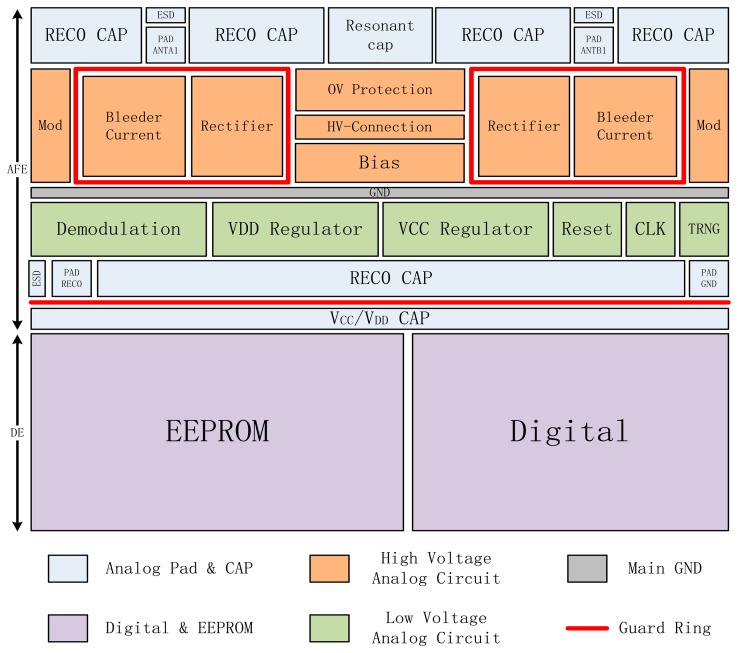
Layout floor plan of the whole chip.

The plan of the whole chip layout is shown in Figure 6. The AFE and DE part of the chip are separated with *V_REC0_* and *V_CC_*/*V_DD_* capacitances so as to isolate noise between them. Since REC0 is designed to be 4 V–12 V, which is much higher than the operation voltage of DE, an NP guard ring is placed between REC0 and *V_CC_*/*V_DD_* capacitances to split the high voltage AFE and low voltage DE areas. Moreover, in the AFE layout, high voltage blocks are apart from low voltage blocks with a main thick ground wire to avoid latch-up. To isolate substrate current in areas with large current flow, an NPN guard ring is added around the current bleeding and rectifier circuits for large currents injected into the substrate that increase the ground voltage which is more likely to lead to a latch-up.

For more considerations, two antenna pads ANTA1 and ANTB1 are placed as mirror images in this chip layout shown Figure 6, as well as wires, devices and circuits connected to the two antennas. Signals transferred through the two antennas are ensured to be symmetrical in this way. Buffer cells are added into the long wires connecting the AFE and DE circuits to suppress signal attenuations.

## 4. Simulation and Measurement Results

The RF powering circuit is designed and simulated in the HJTC 0.25 μm process together with the whole RFID tag. As mentioned in Section 3, PCE of the RF powering circuit designed in HJTC 0.25 μm process is simulated under different load current of *V_CC_*/*V_DD_*. Simulation results are shown in Table 2, PCE of the circuit is 39.17% when the load current is 520 μA while the tag is in its normal working conditions.

Temperature rejection ratio (TRR) and voltage rejection ratio (VRR) are defined as follows:

(16) $$\text{TRR}=\frac{\Delta V_{\text{CC}}}{\text{Temper}}$$

(17) $$\text{VRR}=\frac{\Delta V_{\text{CC}}}{V_{\text{olt}}}$$

where ΔV_CC_ is the variation of regulator output, Temper is the variation range of temperature; V_olt_ is the variation range of rectifier output voltage.

**Figure 7.**
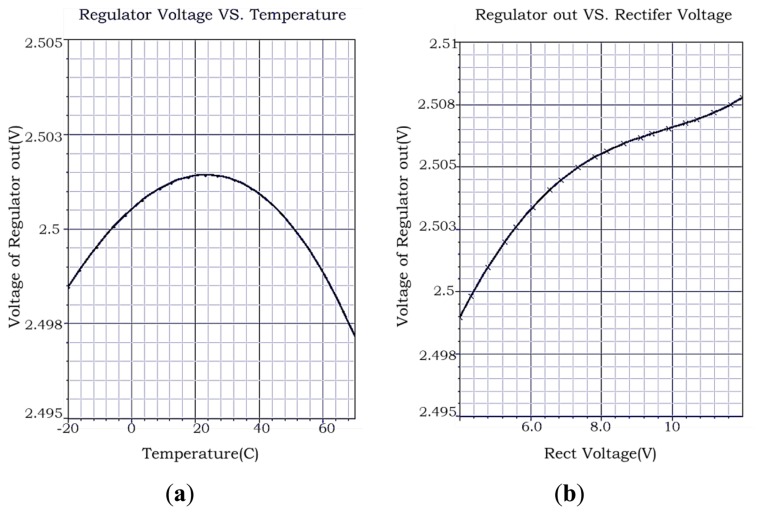
(**a**) *V_CC_ vs.* Temp Simulation results of regulator *V_CC_*, *V_REC0_* = 6V; (**b**) *V_CC_ vs. V_REC0_* Simulation results of regulator *V_CC_*, T = 27 °C.

Shown in Figure 7 are the simulation results of regulator *V_CC_* in the HJTC 0.25 μm process. The calculations of the simulation results are shown in Table 2. The value of PCE can be calculated with the equation PCE = *P_out_*/*P_in_*, while $P_{\text{in}}=\bar{V_{\text{ant}}\cdot I_{\text{ant}}}$ and $P_{\text{out}}=\bar{V_{\text{CC}}\cdot I_{\text{load}}}$, where *V_ant_* is the voltage of the antenna, *I_ant_* is the current of the antenna, *V_CC_* is the output voltage of the regulator, *I_load_* is the load current of the regulator. Considering the usual work environment of the tag, we pay more attention to the value of TRR while *V_REC0_* is near 6 V, and the value of VRR while the temperature is near 27 °C. Under these two conditions the regulator has a quite small TRR and VRR with TRR = 0.04 mV/°C and VRR = 1.1 mV/V.

RFID tags designed in this paper are also measured according to ISO.IEC 15693 standard and related ISO/IEC 10373-7 test standard with contactless test methods. The test electromagnetic field strength (represented as H) defined in the standard is 150 mA/m (*H_min_* = 150 mA/m) to 5 A/m (*H_max_* = 5 A/m), and test simulation signals are in 100% ASK or 10% ASK modulation mode.

**Figure 8.**
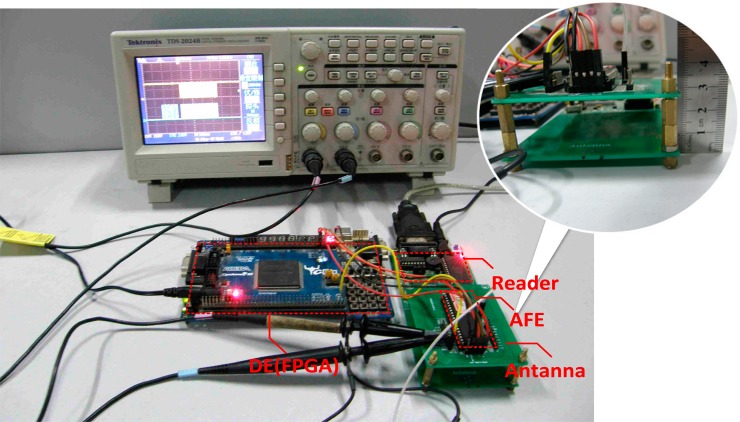
Functional measurement setup based on PCB and the demodulation signal from the tag.

The RF powering circuit has been applied to HF RFID tag chip projects. To make the testing more convenient, the analog front end (AFE) circuit for a HF RFID tag is fabricated, in which this RF powering circuit is integrated. Consisting of analog front end (AFE), digital baseband and EEPROM memory (DE), the RFID tag test platform based on PCB is shown in the Figure 8. The DE part is realized in the form of FPGA, the AFE part is fabricated in the HJTC 0.25 μm process. The RF powering circuit designed above is one part of the AFE in this chip. The partial enlarged view in Figure 8 shows that the distance from reader to the tag can be adjusted to test different electromagnetic field strengths. In Figure 8, the demodulated signal coming from the AFE chip and the return signal which is transformed from the demodulated signal just mentioned via the processing of the DE part are tested by the oscilloscope. In the oscilloscope screen shown in Figure 8, the upper curve is the test waveform of the demodulated signal, while the lower one is the test waveform of the return signal. Both of the test waveforms are normal, which means the communication between the tag and reader is successful. Thus we can affirm that the RF powering circuit can provide voltage source needed by the whole chip.

**Figure 9.**
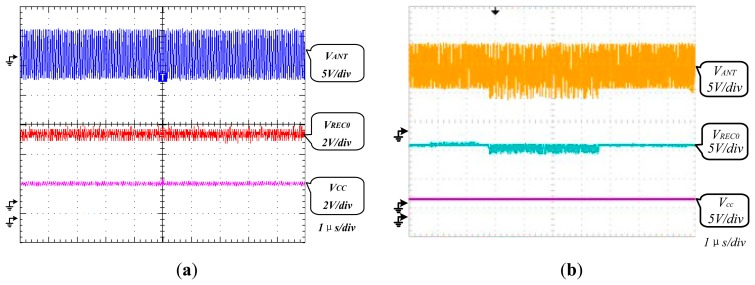
(**a**) *V_CC_* test waveform when H = 0.3 A/m under 0.25 μm process, *V_CC_* = 2.40 V (rms); (**b**) *V_cc_* test waveform when H = 4 A/m under 0.25 μm process, *V_cc_* = 2.42 V (rms).

**Figure 10.**
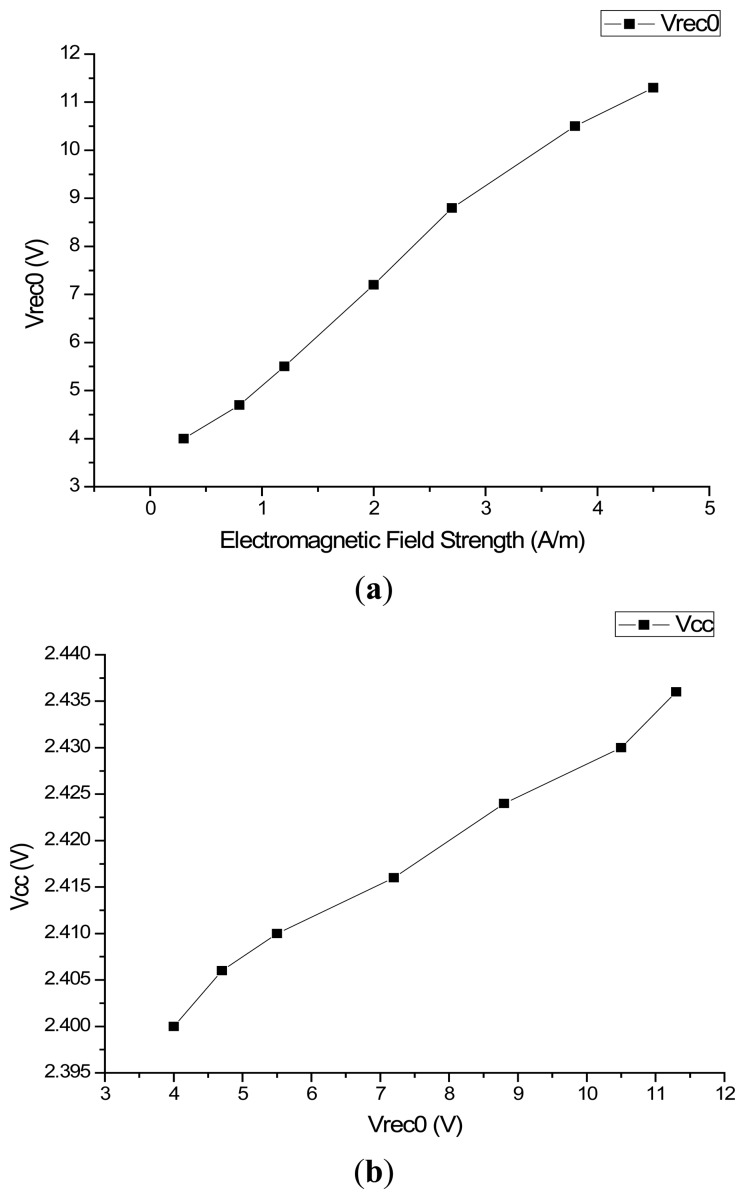
(**a**) *V_rec0_ vs*. Electromagnetic Field Strength; (**b**) *V_cc_ vs. V_rec0_*.

**Figure 11.**
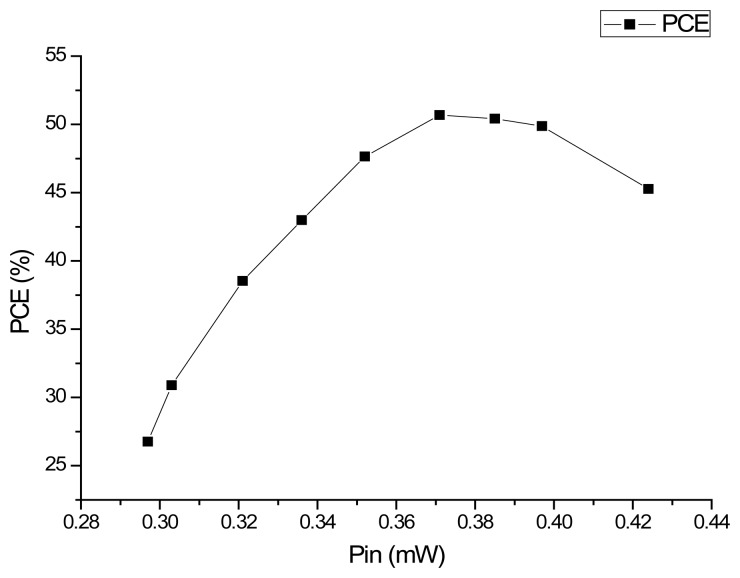
Test results of the PCE of the RF powering circuit in the HJTC 0.25 μm process.

The AFE was tested using a TDS 2024B oscilloscope. The test waveform of V_CC_ is shown in Figure 8. The strength of the electromagnetic field surrounding the RFID tag is adjusted by changing the distance between the chip and reader as shown in Figure 8. The *V_cc_* test waveform when H = 0.3 A/m is shown in Figure 9a, and the one when H = 4 A/m is shown in Figure 9b. Other values of *V_cc_* in different electromagnetic field are also tested, and they are shown in Figure 10. The test results of PCE of this RF powering circuit are shown in Figure 11.

According to Figure 9, the V_CC_ voltage level is 2.4 V within the normal deviation ±10% of 2.5 V. According to Figure 10, considering the usual work environment of the tag, the regulator of this RF powering circuit has a quite small VRR with VRR = 2.5 mV/V while *V_REC0_* is near 6 V. According to Figure 11, considering the usual work environment of the tag, in which the input power of the RF powering circuit is near 0.32 mW, the PCE of the RF powering circuit is 38.54%. Table 3 shows the performance comparison between this RF powering circuit and previous RF powering circuits.

**Figure 12.**
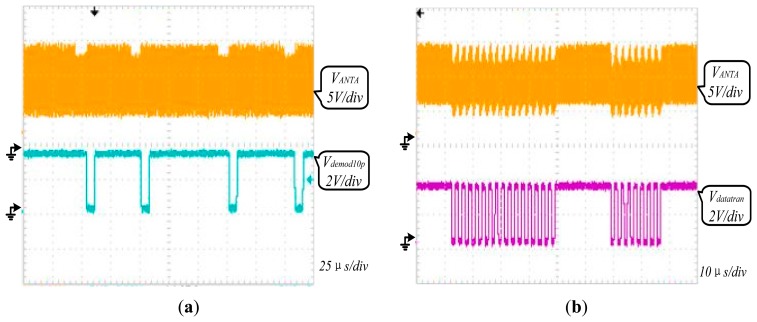
(**a**) Waveform of demodulation; (**b**) Waveform of modulation.

**Figure 13.**
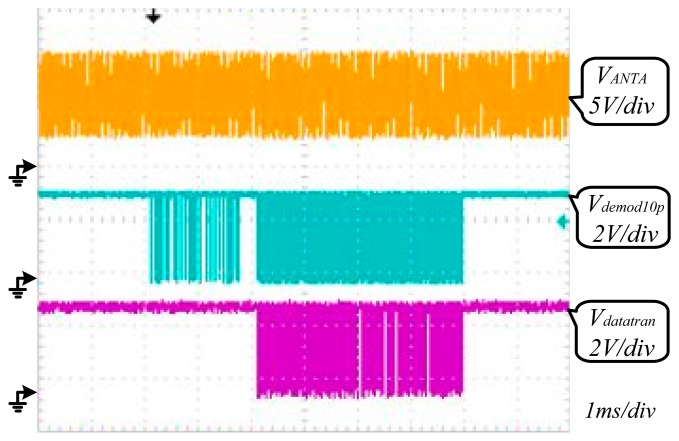
Measured waveform of the communication between reader and tag.

Figure 12a shows that the demodulator of AFE has demodulated the downlink 10% ASK modulated signal sent from the reader successfully. The signal Demod10p is the demodulated signal achieved by the demodulator of AFE. Figure 12b shows an uplink signal of about 13.6% load modulated depth is achieved from the chip antenna to the reader which meets the communication criterion. The signal Datatran determined by the digital baseband controls the AFE modulator. Figure 13 shows that AFE has realized the information communication with reader successfully, which means this RF powering circuit work normally providing a stable voltage source for the whole chip.

**Figure 14.**
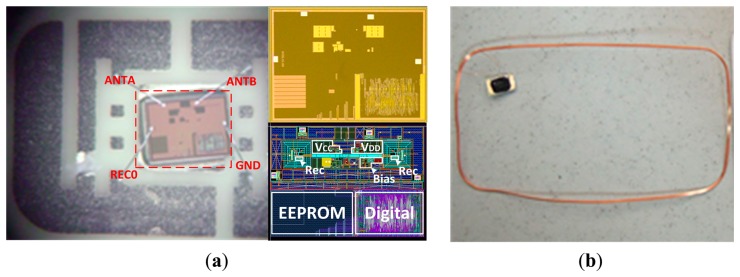
(**a**) Photograph of packaged RFID tag. Chip microphotograph and layout. (**b**) Chip tag used in a RFID smart card.

This RF powering circuit has been integrated in a RFID tag chip. The whole RFID tag packaged, chip die layout and chip photograph are shown in Figure 14. The size of the whole chip is 1.1 × 1.1 mm^2^, whereas the RF powering circuit consumes 0.23 × 0.24 mm^2^ of chip area. In Figure 14, there are also photographs of the packaged RFID tag and the tag used in a RFID smart card. The four pads for the package are two antenna pads and two voltage pads for testing.

## 5. Conclusions

In this work, a RF Powering circuit used for passive RFID tags is designed and fabricated with the HJTC 0.25 μm CMOS process. The circuit consists of a rectifier, a bias circuit and a regulator. It provides two stable power sources for the analog and digital parts, respectively. The RF powering circuit achieves high PCE of 38.54% with the HJTC 0.25 μm process under normal working conditions and the power supply voltage is immune to temperature and voltage variations. Simulation results show that this circuit has a TRR = 0.04 mV/°C, and test results show that it has a VRR = 2.5 mV/V. This RF powering circuit is also fabricated with the HJTC 0.25 μm process in the ISO/IEC15693-Compatible and ISO/IEC 14443-Compatible RFID tag chip. Layout of this chip is optimized for latch-up prevention and noise isolation. Measurement results of the tag show that this circuit can provide a stable voltage source for a HF RFID tag chip for its proper operation.

## Figures and Tables

**Table 1.** Simulation results of NMOS gate cross-connected bridge rectifier (*R_load_* = 6 kΩ, ***V_ANT_*** = 6 V).

*M*	VHD/V	Pin/mW	Pout/mW	PCE
5	2.64	2.60	1.16	44.60%
10	3.01	3.00	1.51	50.23%
20	3.30	3.30	1.83	55.30%
30	3.46	3.53	2.00	56.60%
40	3.55	3.68	2.10	57.05%
50	3.62	3.78	2.18	57.70%

**Table 2.** Simulation results of PCE of RF powering circuit in HJTC 0.25 μm process.

V_REC0_	I_load_	V_ant,p-p_	PCE
4.42 V	800 μA	7.03 V	53.14%
4.88 V	600 μA	7.36 V	43.68%
5.01 V	520 μA	7.43 V	39.17%
5.2 V	400 μA	7.53 V	31.94%

**Table 3.** Comparison with previous RF power circuits.

	This Work	[8]	[14]	[23]	[24]
Process	0.25 μm	0.35 μm	0.18 μm	0.35 μm	0.35 μm
VRR (mV/V)	2.5	39	12	4	0.8
TRR (mV/°C)	0.04	Not Support	0.2	0.01	2
